# Reducing unnecessary antibiotic prescription through implementation of a clinical guideline on self-limiting respiratory tract infections

**DOI:** 10.1371/journal.pone.0249475

**Published:** 2021-04-01

**Authors:** Xavier Sánchez, María Orrico, Toa Morillo, Andrea Manzano, Ruth Jimbo, Luciana Armijos

**Affiliations:** 1 Centro de Investigación para la Salud en América Latina (CISeAL), Pontificia Universidad Católica del Ecuador, Quito, Ecuador; 2 Facultad de Medicina, Postgrado de Medicina Familiar y Comunitaria, Pontificia Universidad Católica del Ecuador, Quito, Ecuador; 3 Universidad Alcalá de Henares, Madrid, España; Rabin Medical Center, Beilinson Hospital, ISRAEL

## Abstract

**Background:**

Clinical guidelines (CG) are used to reduce variability in practice when the scientific evidence is sparse or when multiple therapies are available. The development and implementation of evidence-based CG is intended to organize and provide the best available evidence to support clinical decision making in order to improve quality of care. Upper respiratory tract infections (URTI) are the leading cause of misuse of antibiotics and a CG may reduce the unnecessary antibiotic prescription.

**Methods:**

The aim of this quasi-experimental, before-after study was to analyze the short- and long-term effects of the implementation of a CG to decrease the rate of antibiotic prescription in URTI cases in the emergency department of a third level private hospital in Quito, Ecuador. The study included 444 patients with a main diagnosis of URTI. They were distributed in three groups: a baseline cohort 2011 (n = 114), a first post-implementation cohort 2011 (n = 114), and a later post-implementation cohort 2018 (n = 216). The implementation strategy consisted of five key steps: acceptance of the need for implementation of the CG, dissemination of the CG, an educational campaign, constant feedback, and sustainability of the strategy through continuous training.

**Results:**

The results of this study show a 42.90% of antibiotic prescription rate before the CG implementation. After the implementation of the CG, the prescription rate of antibiotics was significantly reduced by 24.5% (42.9% vs 18.4%, p<0.0001) and the appropriate antibiotic prescription rate was significantly increased by 44.2% (22.4% vs 66.6%, p<0.0001) in the first post-implementation cohort 2011. There was not a significant difference in antibiotic prescription rate and appropriate antibiotic prescription rate between two post-implementation cohorts: 18.4% vs 25.9% (p = 0.125) and 66.6% vs 50% (p = 0.191), respectively.

**Conclusions:**

The implementation of CGs decreases the rate of antibiotic prescription in URTI cases. The results are remarkable after early implementation, but the effect persists over time. The emphasis must shift from guideline development to strategy implementation.

## Introduction

Inappropriate antibiotic prescription practice is among the most commonly discussed public health issues. According to the World Health Organization (WHO), half of all prescribed medications are prescribed in an inappropriate way [1]. Medicines are considered appropriate to be prescribed to the general population when they have a clear, scientific evidence-based indication, are well tolerated in the majority of patients and are cost-effective [2–4]. In addition, a rational use of medications requires that patients receive medications appropriate to their clinical needs.

The common cold is a generic term used to describe a form of mild upper respiratory tract infections (URTIs) caused predominantly by viral pathogens [5]. Actually, the common cold is a heterogeneous group of diseases caused by numerous viruses that belong to several families. However, a viral infection predisposes some patients to bacterial superinfections. About 20–30% of cold symptoms remain without a proven viral cause [6]. This could be explained because of the lack of availability of sophisticated diagnostic methods that can be applied in epidemiological surveys and community-based studies.

The symptomatic treatment of URTI has been aimed at alleviating the most uncomfortable symptoms of the disease. Part of the treatment recommendations about using some medications stem from low-quality studies, so there is variability in treatment among healthcare providers [7]. Although antibiotics are not effective against viruses, they are widely used in the treatment of uncomplicated viral URTI cases [8, 9].

Studies have shown that factors like age, gender, medical specialty, sociodemographic and previous personal experiences can influence a physician’s decision to prescribe antibiotics in primary care [10]. The prescription of an antibiotic is influenced by the patient’s demand and expectations, the health care provider’s knowledge of evidence-based medicine, current guidelines, years of professional experience, lack of knowledge about the proper use of antimicrobials, complacency with the patient, provider’s fear to fail to treat the patient’s illness, and lack of time or availability of drugs [11]. Thus, the prescription of an antibiotic is influenced by factors that affect all stakeholders, including physicians, other health care providers, the health system, and patients. These factors are mutually related [12]. On the other hand, factors related to symptoms found in physical exams such as fever, purulent sputum, abnormal respiratory exam, and tonsillar exudate, have also been associated with antibiotic prescription in URTI cases; as health care providers believe that they are more indicative of a bacterial etiology [13].

Various strategies have been proposed to reduce inappropriate antibiotic prescription in URTI cases. The most studied interventions are educational materials for physicians, audits and feedback, educational meetings, changes in the financial and healthcare systems, reminders, electronic assistance systems, patient-target interventions, and multifaceted physician-target interventions [14–16]. Among all the interventions, those that include educational material for doctors and parents, were the most effective in reducing the use of antibiotics in URTI cases.

Another strategy used to reduce the use of antibiotics is to delay the prescription of antibiotics. Different methods of delaying prescriptions (such as giving prescriptions with instructions, leaving prescriptions for collection, post-dating prescriptions, or requesting recontact) have been used [17]. With the strategy of delaying antibiotic prescriptions, less than 40% of patients are likely to use antibiotics [18, 19].

Clinical guidelines (CG) can be defined as “any document containing recommendations for clinical practice”, that are systematically developed in order to assist decisions about appropriate health care for specific clinical circumstances [20, 21]. CG are used to reduce variability in practice when the scientific evidence is sparse or when multiple therapies are available. The development and implementation of evidence based CG is intended to organize and provide the best available evidence to support clinical decision-making in order to improve quality of care, patient outcomes, and cost-effectiveness [22]. CG have been shown to be effective tools to improve the appropriate use of antibiotics in hospital settings [23–25]. Taking into account the evidence published to date, researchers consider that the implementation of CG could reduce the prescription of antibiotics in URTI cases in primary care setting.

## Materials and methods

Ethics approvals for the protocol and the study were granted by the Subcommittee for Research Ethics on Human Beings–PUCE with authorization code SB-CEISH-POS-93. The Ethics Committee established the non-need for informed consent for this study.

### Study design and population

The aim of this quasi-experimental, before and after study, was to analyze the short- and long-term effects of the implementation of CG at a health care facility, and to decrease the rate of antibiotic prescription in URTI cases. The CG were implemented in the emergency department of a specialized private health care facility (third-level hospital) in Quito, Ecuador. The study’s population consisted of patients from three moments in time, a baseline and two post-implementation cohorts. All of these consisted of patients registered in the hospital’s health records (HR) with a primary diagnosis of URTI according to The International Classification of Disease (ICD) 10 codes, Table 1. The baseline was measured from January 1, 2010 through March 31, 2010. The implementation process of the CG was from May 1, 2010 through December 31, 2010. Post-implementation was measured from January 1, 2011 through March 31, 2011, and 7 years later, from January 1, 2018 through December 31, 2018.

**Table 1:** ICD-10 codes considered as URTI for the purpose of this study.

• J00 Acute nasopharyngitis [common cold] • J01 Acute sinusitis *(includes J01*.*0*, *J01*.*1*, *J01*.*2*, *J01*.*3*, *J01*.*4*, *J01*.*8*, *J01*.*9)* • J02 Acute pharyngitis *(includes J02*.*0*, *J02*.*8*, *J02*.*9)* • J03 Acute tonsillitis *(includes J03*.*0*, *J03*.*8*, *J03*.*9)* • J04 Acute laryngitis and tracheitis *(includes J04*.*0*, *J04*.*1*, *J04*.*2)* • J05 Acute obstructive laryngitis [croup] and epiglottitis • J06 Acute upper respiratory infections of multiple and unspecified sites *(includes J06*.*0*, *J06*.*8*, *J06*.*9)* • J10 Influenza due to other identified influenza virus *(includes J10*.*1)* • J11 Influenza due to unidentified influenza virus *(includes J11*.*1)*
Following codes also were considered as URTI, diseases of the middle ear categorized according to ICD-10 as:
• H65 Nonsuppurative otitis media • H66 Suppurative and unspecified otitis media

ICD: International Classification of Disease, URTI: Upper respiratory tract infection

### Intervention

Researchers considered the intervention as the introduction process of the CG, which included different phases. The CG that were chosen for implementation, considering the lack of National CG for URTI cases in Ecuador, were the Clinical Guidelines CG69, “Respiratory Tract Infections (self-limiting): prescribing antibiotics” by the National Institute for Health and Care Excellence (NICE) [26]. The guidelines provide information on the diagnosis and treatment of URTI cases and offer prescription recommendations for antibiotics, such as for clinically compromised patients or patients with comorbidities, who are at high risk of bacterial superinfection. The CG propose practical strategies for prescribing antibiotics in children and adults, such as: 1. Immediate antibiotic prescription, 2. Delayed antibiotic prescription and 3. No antibiotic prescription. Table 2 describes the criteria for cases that need antibiotic prescription for each ICD-10 category, according to the NICE CG.

**Table 2:** Need for prescription of antibiotics in URTI according to the guideline using ICD-10 codes.

ICD-10 code	Criteria for Appropriate Antibiotic Prescription
**H65** Nonsuppurative otitis media **H66** Suppurative and unspecified otitis media	**H65** must meet both conditions: presence of Acute Bilateral Otitis media AND age <2 **H65** or **H66** with presence of otorrhea
**J01** Acute sinusitis	Must meet all conditions: Fever of >38C°, purulent discharge and facial pain
**J02** Acute pharyngitis **J03** Acute tonsillitis	Must meet **3 of the following CENTOR criteria**: • Presence of tonsillar exudate • Presence of painful anterior cervical lymphadenopathy or lymphadenitis • Fever (>38°C) • Absence of cough
**J00** Acute nasopharyngitis [common cold] **J01** Acute sinusitis **J02** Acute pharyngitis **J03** Acute tonsillitis **J04** Acute laryngitis and tracheitis **J05** Acute obstructive laryngitis [croup] and epiglottitis **J06** Acute upper respiratory infections of multiple and unspecified sites **J10** Influenza due to other identified influenza virus **J11** Influenza due to unidentified influenza virus	**Must meet any** of the following criteria: • Presence of one or more of the following comorbidities: Cardiac, Pulmonary, Renal, Hepatic, Neuromuscular, Immunosuppression, Cystic Fibrosis, Diabetes Mellitus. **OR** • Age <2 years old **AND** history of prematurity **OR** • Age >65 years old **AND** presence of cough **AND two or more** of the following: ◾ Hospitalized (recent) ◾ Diabetes mellitus ◾ History of cardiac arrest ◾ Current use of corticosteroids **OR** • Age >80 years old **AND** presence of cough **AND one or more** of the following: ◾ Hospitalized (recent) ◾ Diabetes mellitus ◾ History of cardiac arrest ◾ Current use of corticosteroids

URTI: Upper respiratory tract infection, ICD: International Classification of Disease, there are no ICD-10 codes for J07 and J08. J09 excluded because it refers to influenza

The implementation of the CG consisted in five different phases:

1. An explanation of the need for rational antibiotic prescription practices and guidance to the main stakeholders (hospital authorities and all clinicians that work in the emergency department) involved with antibiotic prescription problems at different hospital levels was carried out. The objective was to increase awareness about the adequate prescription of antibiotics in URTI cases and the possibility of reducing the use of antibiotics through the implementation of clinical guidelines. This was followed by the acceptance of the implementation by all physicians.
2. Diffusion of the guidelines by distribution of hard copies of the CG to all clinicians was performed. The copies were also sent via electronic mail, along with relevant background information that could help clinicians apply the CG.
3. An educational campaign was furtherly carried out by experts on the field: three family physicians and one infectious disease specialist, to improve knowledge of URTIs, focusing on improving the correct diagnosis and reducing the use of antibiotics. Posters with treatment algorithms were published in work areas of healthcare providers and nursing stations around the hospital. A total of five training sessions were provided in the implementation period. The training sessions lasted two hours and the teaching strategy was applied through educational games (like jeopardy games), leaflet distribution and pocket leaflets. Patient information sheets were also available for distribution; in this way, clinicians could provide the patients with URTI cases with general recommendations and align their prescriptions with the guideline recommendations.
4. Consistent feedback from the implementation team to physicians was also applied to reinforce proper management and treatment of URTI cases through individual audit sessions each week during the implementation period.
5. Guarantee of sustainability through continuous training was the last phase of implementation. The clinicians committed to continue the dissemination of the CG and the educational campaign every year to all clinical staff in the emergency department and whenever there would be a new staff member in the department.

### Sample

The sample was defined as all the patients that met the inclusion criteria and that had complete information in their HR. A simple probabilistic type sampling was performed. The sample for the baseline and two implementation period cohorts consisted of patients with a main diagnosis of URTI according to ICD-10 codes in the emergency department. The following formula was applied to calculate the sample for a finite universe, n = N*Z^2^*p*q / d^2^*(N-1) + Z^2^*p*q, in each period of time.

In this formula, *N* represents population size, *Z* is the confidence level (95%), *p* is the probability of success, or expected proportion (50%), *q* is the probability of failure (50%), and *d* is precision (3% of maximum admissible error in terms of proportion). We decided to increase the sample by 10% considering possible losses. The subsequently studied sample comprised 114 HR for baseline, 114 HR for post-implementation cohort 2011 and 216 HR for post-implementation cohort 2018.

HR were selected according to the sample number among all cases of URTI registered in the emergency department. The selection of HR was randomized through a computer software that threw random numbers automatically (Epidat 4.1 version statistical software). None of the HR were excluded, as they had all the required information properly recorded.

### Data source and data collection

In order to analyze the effect of the intervention in reducing antibiotic prescription, data from the HR from all the patients included in the three different cohorts mentioned was used. The collected data comprised the patient’s age, sex, clinical presentation, and presence of comorbidities related to the criteria for prescribing antibiotics according to the CG, Table 2. A follow-up of the patients was performed until the moment of the clinical discharge of the URTI episode (including any visits of the patient to any other outpatient department). Complications were considered for this study as the need for hospitalization for any reason related to the primary diagnosis of URTI and the subsequent need for antibiotics during patient follow-up. Data were anonymously and manually extracted from the HR simultaneously by two peer reviewers, according to the following criteria:

- Inclusion criteria: Patients 3 months of age and above who required clinical ambulatory care for URTIs in the emergency department.
- Exclusion criteria: Patients whose primary diagnosis of URTI was determined by another outpatient department of the hospital.

After individual data extraction, the information was compared, and a consensus of inclusion or exclusion was reached for each patient. Two types of health professionals worked in the designated department and diagnosed the patients with URTI that were included in the study. These health professionals are classified as:

- Family Medicine Doctor: Medical specialist who has completed a 3-year postgraduate degree in general medicine.
- Emergency physician: Medical specialist who has completed a 3 to 4-year postgraduate degree in Emergency Medicine.

The need for antibiotic prescription was assessed according to the recommendations in the CG. The criteria for justified prescription of antibiotics are shown in Table 2. The HR were evaluated by two independent reviewers (medical specialists in primary care) and when there was inconsistency between the reviewers, this was resolved by consensus.

### Antibiotic prescription evaluation

The antibiotic prescription rate was defined as the number of antibiotic prescriptions divided by all patients diagnosed with URTI, and appropriate prescription rate as the number of appropriate antibiotic prescriptions according to the NICE CG recommendations, divided by all patients receiving antibiotics.

### Statistical analysis

The variables included in this study were both categorical and quantitative. The researchers performed a descriptive analysis with categorical variables through frequency distributions, proportions, and rates, and an analysis of quantitative variables through measures of central tendency and dispersion. The differences between the proportions of the variables in the cohorts (baseline, and post-implementation periods) were evaluated using the z test (*t-test* for independent proportions), where p<0.05 was considered significant. Epidat 4.1 version statistical software was used for data analysis.

## Results

The general characteristics of the patients are described in Table 3.

**Table 3:** Characteristics of the sample.

Characteristic	Baseline 2011 n = 114	Post-implementation 2011 n = 114	p value	Post-implementation 2018 n = 216	p value
**Age Mean** (SD)	22.98 (21.07)	25.35 (20.42)	0.389	33.37 (28.05)	0.003
**Gender**					
Male	56 (49.12)	51 (44.73)	0.507	96 (44.44)	0.959
Female	58 (50.87)	63 (55.26)	0.507	120 (55.55)	0.959
**Diagnosis**					
Acute tonsillitis	23 (20.17)	4 (3.5)	<0.0001	19(8.79)	0.073
Acute pharyngitis	15 (13.15)	8 (7)	0.12	55 (25.46)	<0.0001
Acute nasopharyngitis	53 (47.49)	47 (41.2)	0.838	68 (31.48)	0.077
Acute laryngitis	13 (11.40)	14 (12.3)	0.838	1 (0.46)	<0.0001
Acute sinusitis	4 (3.50)	4 (3.50)	1	11 (5.09)	0.511
Acute bronchitis	3 (2.63)	5 (4.40)	0.472	36 (16.66)	0.001
Acute otitis media	2 (1.75)	6 (5.26)	0.150	8 (3.70)	0.504
Influenza	1 (0.87)	26 (22.80)	<0.0001	18 (8.33)	<0.0001
**Comorbidity**	4 (3.50)	3 (2.63)	0.701	4 (1.85)	0.640
**Health professional**					
Emergency	75 (65.78)	81 (71.050	0.393	139 (64.35)	0.219
Family Medicine	39 (34.21)	33 (28.94)	0.393	77 (35.64)	0.219

Data are presented as number (percentage) of patients except where noted, SD: Standard deviation

The study included 444 patients with a main diagnosis of URTI that met the inclusion criteria. They were distributed in three groups: i) a baseline cohort (n = 114), ii) a first post-implementation cohort 2011 (n = 114), and iii) a later post-implementation cohort 2018 (n = 216). Overall, patients in all three time periods had similar demographic characteristics.

Acute nasopharyngitis (common cold) was the most common diagnosis among the baseline and the post-implementation cohorts of 2011 and 2018, representing 47.4%, 41.2% and 31.48%, respectively. Amid the three periods, most of the diagnoses were made by emergency physicians: 65.8%, 71.1% and 64.35%, respectively.

There was a significant difference between the baseline and the post-implementation cohort of 2011 in the diagnosis of acute tonsillitis (20% vs 3.5%; p<0.0001), and in the diagnosis of influenza (0.87% vs 22.8%, p<0.0001). Differences between the post-implementation cohort of 2011 and the post-implementation cohort of 2018, were significative in the diagnosis of acute pharyngitis (7% vs 25.4%, p<0.0001), acute laryngitis (12.3% vs 0.46%, p<0.0001) and influenza (22.8% vs 5.09%, p<0.0001).

### Antibiotic use

Broad spectrum antibiotics were used in all patients during the three periods, as shown in Table 4. Azithromycin and penicillin G benzathine were the most prescribed antibiotics in the baseline cohort (36.73% and 24.48%, respectively). Amoxicillin-clavulanate and penicillin G benzathine were the most prescribed antibiotics in the post-implementation cohort of 2011 (42.85% and 19.04%, respectively) and in the post-implementation cohort of 2018 (37.5% and 17.85%, respectively). There was a significant reduction in the use of azithromycin between the baseline period and the post-implementation cohort of 2011 (36.73 vs 9.52%, p = 0.021).

**Table 4:** Antibiotic use.

Antibiotic	Baseline 2011	Post-Implementation 2011	*p* Value	Post-Implementation 2018	*p* Value
Azithromycin	18/49 (36.73)	2/21 (9.52)	0.021	9/56 (16.07)	0.465
Amoxicillin	2/49 (4.08)	2/21 (9.52)	0.369	6/56 (10.71)	0.879
Cefuroxime	6/49 (12.2)	2/21 (9.52)	0.743	4/56 (7.14)	0.728
Cephalexin	1/49 (2.04)	0/21 (0)	0.053	3/56 (5.35)	0.917
Clarithromycin	0/49 (0)	2/21 (9.52)	0.15	3/56 (5.35)	0.509
Penicillin G Benzathine	12/49 (24.48)	4/21 (19.04)	0.619	10/56 (17.85)	0.904
Amoxicillin clavulanate	10/49 (20.40)	9/21 (42.85)	0.053	21/56 (37.50)	0.668

Data are presented as number (percentage) except where noted

### Antibiotic prescription rate and appropriate antibiotic prescription rate

Antibiotic prescription rates in the post-implementation cohort of 2011 were significantly reduced when compared with the prescription rates in the baseline. Antibiotic prescriptions decreased by 24.5% (42.9% in the baseline vs 18.4% in the post-implementation cohort of 2011, p<0.0001). There was not a significant difference in antibiotic prescription rates between the two post-implementation periods (18.4% vs 25.9%, p = 0.125), (Table 5). Appropriate antibiotic prescription rates between the baseline and the post-implementation cohort of 2011 were significantly increased by 44.2% (22.4% vs 66.6%, p<0.0001). There were no significant differences in appropriate antibiotic prescription rates between the two post-implementation periods (66.6% vs 50%, p = 0.191), as shown in Table 5.

**Table 5:** Antibiotic prescription and appropriate antibiotic prescription.

Variable	Baseline 2011	Post-Implementation 2011	*p* Value	Post-Implementation 2018	*p* Value
Antibiotic prescription	49/114 (42.98)	21/114 (18.42)	<0.0001	56/216 (25.92)	0.125
Appropriate prescription	11/49 (22.44)	14/21 (66.66)	<0.0001	28/56 (50.00)	0.191

Data are presented as number (percentage) of patients except where noted.

Differences in antibiotic prescription rates in children and adults are shown in Table 6. There was a significant reduction of 31.6% (*p*<0.001) in the antibiotic prescription rate in the group of adults after the early implementation, but no differences were found in the children group. There were no differences between the two post-implementation periods.

**Table 6:** Antibiotic prescription rates in children and adults.

Variable	Baseline 2011	Post-Implementation 2011	*p* Value	Post-Implementation 2018	*p* Value
Children	20/53 (37.73)	10/45 (22.22)	0.097	21/90 (23.33)	0.885
Adults	29/61 (47.54)	11/69 (15.94)	<0.001	35/126 (27.77)	0.063

Data are presented as number (percentage) of patients except where noted.

Diagnosis-specific antibiotic prescription rates are shown in Table 7. After the early implementation, there was a reduction of 18.4% (p = 0.008) in antibiotic prescription rates when the diagnosis was acute nasopharyngitis, but there were no significant differences between the two post-implementation periods. For the rest of diagnoses, antibiotic prescription rates were similar among different periods. In the cases where there was a decrease in antibiotic prescription, there were not any medical complications reported during either of the three periods.

**Table 7:** Diagnosis-specific antibiotic prescribing rates.

Diagnosis	Baseline 2011 n = 114	Post-implementation 2011 n = 114	p value	Post-implementation 2018 n = 216	p value
Acute tonsillitis	20/23 (86.95)	4/4 (100)	0.687	11/19 (57.89)	0.307
Acute pharyngitis	6/15 (40.00)	2/8 (25.00)	0.472	10/55 (18.18)	0.646
Acute nasopharyngitis	12/53 (22.64)	2/47 (4.25)	0.008	5/68 (7.35)	0.495
Acute laryngitis	4/13 (30.76)	3/14 (21.42)	0.580	1/1 (100)	0.347
Acute sinusitis	4/4 (100)	3/4 (75.00)	0.621	9/11 (81.81)	0.770
Acute bronchitis	1/3 (33.33)	4/5 (80.00)	0.187	14/36 (38.88)	0.083
Acute otitis media	2/2 (100)	2/6 (33.33)	0.343	4/8 (50.00)	0.533
Influenza	0/1 (0)	1/26 (3.84)	0.056	2/18 (11.11)	0.056

Data are presented as number (percentage) except where noted

## Discussion

The implementation of CG requires changes in the attitudes and behavior of health professionals as well as adaptations of the structural environment [27, 28]. We implemented CG in order to reduce antibiotic prescription rates in a common group of related diseases treated in ambulatory settings.

The results of this study show an antibiotic prescription rate of 42.90% before the CG implementation. This number turned out to be even higher than the one obtained by our previous study in Ecuador from 2015, where the rate of prescription for antibiotics in URTI cases in an ambulatory care center was 37.5% [29]. Studies from all over the world have estimated an average of around 50% [30–37] of antibiotic prescriptions in URTI cases. Cordoba, et. al published a study in 2016 [38] involving four Latin-American countries; said study evaluated antibiotic prescription practices in URTI diagnoses in primary care health centers. The rates of antibiotic prescription were: 40% in Bolivia, 35% in Argentina, 27% in Uruguay, and 24% in Paraguay. In Mexico, Doubova et al. [39] reported more than 61% of antibiotic prescriptions in children diagnosed with non-streptococcal URTI after a first visit to the health facility. Even if these studies did not evaluate the appropriate prescription rate, the results are comparable to the current research.

An appropriate antibiotic prescription rate of 22.4% was found before CG implementation, reflecting an inappropriate prescription rate of almost 80%. A study done by Bagger et al. [8] in Argentina, Denmark, Lithuania, Russia, Spain, and Switzerland reported a 50% of inappropriate antibiotic prescription rates in URTI and almost 100% of inappropriate antibiotic prescriptions for common cold and otitis media. Holloway et al. [40] investigated the treatment of childhood infections in 78 lower-middle income countries between 1990 and 2009; after the review of 344 studies, the results showed a high percentage of URTI cases treated with antibiotics, with this percentage increasing over time (from 42% before 1990 up to 72% in 2006–2009). The study reports 25.8% of inappropriate antibiotic use in URTI cases for Latin America and 47.1% in lower-middle income countries in general. These studies show a comparable inappropriate antibiotic prescription rate in URTI cases to the one found in this paper.

Our results demonstrate that after the implementation of the CG, the prescription rate of antibiotics was significantly reduced by 24.6% in all patients. This result is similar to that reported in a study conducted in the United States [41], in which the strategy included CG implementation aimed to reduce the use of antimicrobials for the treatment of URTI cases in adult and pediatric patients in four primary care clinics. The intervention consisted of half-day educational sessions, delivery of CG, and case study presentations to review the appropriate URTI treatment and diagnosis. After the implementation, there was a significant reduction in the rate of antibiotic prescription by 24.6% in all physicians. In a study from Thailand [42] that determined the effectiveness of the implementation of CG in prescribing antibiotics for adults with URTI, there was a significant reduction of 29.9% in the antibiotic prescription rate. The results from this study are almost identical to ours (antibiotic prescription rate in adults reduced by 31.6%). The implementation strategies applied were educational interventions through two sessions of interactive educational meetings during a month and the distribution of a simple one-page clinical practice protocol. This study showed that strategies that involve educational campaigns, feedback of cases and educational material (e.g. clinical guidelines) in the implementation process are effective. Systematic reviews where physician-targeted multiple interventions or multifaceted interventions containing at least educational material proved effective in reducing antibiotic prescription rates in URTI cases [15, 43].

A significant reduction in the diagnosis of acute tonsillitis was found after early implementation. This result can be attributed to a better diagnosis in URTI cases made by physicians, as this is one of the objectives of the NICE guideline. Nevertheless, despite this reduction, the antibiotic prescription rate in acute tonsillitis remained high. On the other hand, the diagnosis of acute nasopharyngitis (common cold) was the same among different periods, but antibiotic prescription rate was significantly reduced for this condition.

Even though antibiotic prescription rates in children were reduced according to the results, this was not statistically significant. This could be because a low antibiotic prescription rate in children (37.7%) was found in our sample. International data report a global antibiotic prescription rate of 47.3% [44] in URTI cases in children; countries like Italy and Canada have higher rates (42% - 57%) and the Netherlands and the United Kingdom have lower rates (14% - 21%). We hypothesize that the implementation of CG to reduce the prescriptions of antibiotics in children would be effective in settings with higher rates of antibiotic prescription.

### Implications for practice

CGs are relevant tools which should be implemented in order to reach better outcomes in health care. CGs are one important piece of the larger evidence-based practice actions needed to provide better health care. Despite all initiatives to develop such tools, their uptake in practice is not apparent. Developing good quality CGs does not ensure that the recommendations will be implemented in healthcare practice; therefore, the final effect in real practice is never guaranteed. As a matter of fact, the emphasis must shift from guideline development to strategy of implementation [45, 46]. Specific strategies designed to handle possible obstacles during implementation are needed to improve adherence to recommendations. A comprehensive strategy to disseminate the CG appears to be very important, followed by constant support in the learning process as well as a well-designed and well-prepared implementation process [47].

### Limitations of the study

Some limitations can be identified in this study. The results are subjected to period effect, even though we analyzed three different time periods (one baseline and two post-implementation periods). A design that includes post-implementation analyses for each year would be more precise in measuring the change of patterns in antibiotic prescription practice. Additionally, the sample size of the baseline of the study was calculated a priori, but the defined time period was chosen based on the researcher’s convenience, which could have led to selection bias. However, in any case, this limitation would in fact diminish the effect of CG implementation in the long term, but the results have been maintained. We believe that generalization of the results is limited because the implementation of the CG was carried out in a single private institution, although similar patterns in antibiotic prescription have been described in different scenarios [48].

Another limitation of this study is the lack of a National Clinical Guideline for URTIs, provoking a consideration for the use of an international guideline from a source that has been previously used by the Ministry of Public Health in Ecuador. A further limitation that can be mentioned is the lack of adoption of a delayed prescription strategy by physicians which should be included in the CG. This can be explained because of the context in an emergency department as compared to that of an outpatient clinic, making it complicated for patients to request recontact or for physicians to leave prescriptions for later collection; however, patient follow up was performed on the patients and there was no subsequent need for antibiotics in the post-implementation periods.

### Strengths of the study

The strength of the study is found within the methodology for the implementation process. Different levels were included to make a proper implementation process. The success of the CG implementation depended on the consideration of different barriers (related to physician factors, guideline related factors, and external factors) and the use of appropriate strategies to overcome them [20]. The participation of all clinical staff and stakeholders in management was necessary in order to generate awareness and commitment at the provider level as well as support at the administrative level. The dissemination of the CG was extensive and key recommendations were also provided to promote familiarity with the information, generating positive expectations about the plausibility of the recommendations. In addition, the study provided accompaniment in the learning process through educational campaigns, with future reinforcement of what had been learned and positive individualized feedback during training and in practice. We believe that all of these strategies were properly implemented and prompted the success of our intervention.

### Implications for future research

Given the success of the implementation of the CG to reduce unnecessary antibiotic prescription in URTI cases in the study, the proposed intervention might be applicable for other common diseases in which prescription is necessary to improve the health of the patient. Considering that the implementation of a CG does not require a significant time commitment or significant financial resources, future studies could be carried out to identify the real effect of this strategy to improve prescription practices.

## Conclusions

Implementation of CG to reduce unnecessary antibiotic prescription on self-limited URTI cases was effective. The results are remarkable after the early implementation, but the effect can only persist during time as long as the strategy of implementation not only includes aspects related to the guideline but also aspects related to stakeholders, medical staff, and accompaniment in the learning process.

## Supporting information

S1 Data (XLSX)

S2 Data (XLSX)
